# Survivorship and prognostic factors for pleomorphic liposarcoma: a population-based study

**DOI:** 10.1186/s13018-021-02327-3

**Published:** 2021-03-04

**Authors:** Lu Wan, Chao Tu, Lin Qi, Zhihong Li

**Affiliations:** 1 Department of Orthopedics, The Second Xiangya Hospital, Central South University, 139 Renming Road, Changsha, 410011 Hunan People’s Republic of China; 2 Vaccine and Immunotherapy Center, Infectious Diseases Division, Department of Medicine, Massachusetts General Hospital and Harvard Medical School, 149 13th Street, Charlestown, MA 02129 USA; 3 Key Laboratory of Tumor Models and Individualized Medicine, The Second Xiangya Hospital, Central South University, 139 Renming Road, Changsha, 410011 Hunan People’s Republic of China

**Keywords:** Pleomorphic liposarcoma, SEER, Disease-specific survival, Prognosis

## Abstract

**Background:**

Pleomorphic liposarcoma is the least common but most aggressive subtype of liposarcoma. Very few studies have presented data on pleomorphic liposarcoma specifically, often including a limited number of cases and short-term follow-up. As a result, the survivorship and prognostic characteristics of this tumor remain incompletely identified.

**Study design and setting:**

Cross-sectional analysis of the Surveillance Epidemiology and End Results database (1996–2015).

**Results:**

Overall survival for the entire series was 54% (95% confidence interval [CI], 49–58%) and 40% (95% CI, 35–45%) at 5 and 10 years, respectively. Disease-specific survival for the entire series was 60% (95% CI, 56–65%) and 53% (95% CI, 48–58%) at 5 and 10 years, respectively. Patients who survived 10 years or more were more likely to die of events unrelated to pleomorphic liposarcoma. Univariate and multivariate analysis demonstrated that not receiving cancer-directed surgery was an independent poor prognostic factor. Older age (≥ 65 years old) was associated with worse overall survival but not disease-specific survival. Tumor stage and radiotherapy showed different impact on survival depending on tumor size. In comparison to localized staged tumors, regional stage only predicts poor survival in patients with tumor size less than 5 cm, while distant stage is an independent worse prognosis factor. Radiotherapy only benefits patients with tumor size larger than 10 cm. These results were confirmed in competing risk analysis.

**Conclusion:**

Survival rates of patients with pleomorphic liposarcoma has not changed over the past 20 years. Patients with distant stage have poor prognosis; regional stage indicates worse survival in patients with tumor size less than 5 cm. Receiving surgery could prolong the survival, while radiotherapy only benefits patients with large tumor size (> 10 cm). Older age is associated with poor overall survival but not disease-specific survival. Routine patient surveillance following initial diagnosis should at least be 10 years for pleomorphic liposarcoma.

**Supplementary Information:**

The online version contains supplementary material available at 10.1186/s13018-021-02327-3.

## Introduction

Pleomorphic liposarcoma is a rare malignancy that represents approximately 5–10% of all liposarcomas [1–4]. Previously reported 5-year survivorship of pleomorphic liposarcoma was ranging from 29 to 63% [4–9], a figure that is significantly worse than other forms of liposarcoma [3, 5, 10] and which more parallel to other high-grade soft tissue sarcomas such as leiomyosarcoma and myxofibrosarcoma [7, 11].

Identifying survival and prognostic factors of a specific disease is valuable for modern clinical practice. Previous reports describing the outcomes of patients with pleomorphic liposarcoma have limited statistical power owing to the relatively small cohorts of patients [6–9, 12]. To date, only three large series reported by Kimberly et al. [4] in 2006, Gebhard et al. [7] in 2002, and Hornick et al. [8] in 2004 included 64, 63, and 57 cases from the Memorial Sloan-Kettering Cancer Center, French Federation of Cancer Centers, St. Thomas’ Hospital and Brigham and Women’s Hospital, respectively; while other reports included cases are less than 30 patients [5, 6, 9, 12]. Thus, a population-based study is more likely able to provide a comprehensive understanding of the survival and prognostic factors for this rare tumor.

To date, thousands of studies based on the SEER (Surveillance, Epidemiology and End Results) database have been performed to evaluate the outcomes of various type of cancer [13–15], and it also serves as the most frequently used and best estimate of cancer incidence in the USA [16–18]. Given its population-based advantage, SEER database also gives us chance to get a clear perception of some rare diseases. Herein, we present for the first time the SEER data on pleomorphic liposarcoma.

We asked: [1] What are the clinical characteristics of patients diagnosed with pleomorphic liposarcoma? [2] What is the 5- and 10-year survival rate of patients diagnosed with pleomorphic liposarcoma? [3] How long is the appropriate follow-up time for a patient diagnosed with pleomorphic liposarcoma? [4] What is the effect of demographics, tumor characteristics, and treatment options on survival in patients diagnosed with pleomorphic liposarcoma?

We present the following article in accordance with the STROBE reporting checklist [19].

## Methods

### Study design and setting

Cross-sectional analysis of the Surveillance Epidemiology and End Results database (1996–2015).

### Patient selection

**Fig. 1:**
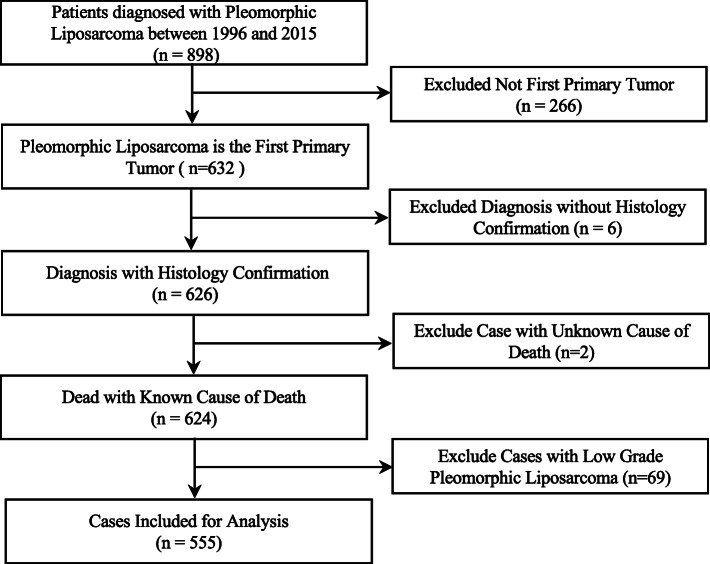
Data extraction flow chart

The latest SEER data, based on the April 2019 release, was used to identify all cases of pleomorphic liposarcoma that were diagnosed between 1996 and 2015 with the use of the ICD-O-3 (International Classification of Diseases for Oncology, Third Edition) morphology codes 8854/3. The inclusion criteria were patient with pleomorphic liposarcoma as the first primary tumor, diagnosed with histology confirmation, and with a known cause of death. Due to pleomorphic liposarcoma is considered as high grade (grades III/IV, poorly differentiated or undifferentiated) in almost all cases, we also excluded cases in low grade (grades I/II, well or moderately differentiated) to avoid potential bias. Data on a total of 555 patients meet the inclusion criteria and were extracted from the database (Fig. 1). The median follow-up of the entire series was 100 months. Two of the authors (LW and CT) independently reviewed the collected data to ensure adequate quality, when there was a discrepancy, these two authors cross reviewed and reached an agreement before conducting analyses.

As previously described [20], we chose the SEER database because SEER is the only comprehensive source of population-based information in the USA that contains stage, size grade of tumor at the time of diagnosis, and patient survival data; currently, it covers approximately 30% of the US population, thus encompassing a substantial sample size that would not otherwise be possible with any single or multi-institutional experiences. Although the SEER database has several shortcomings, including limited number of variables available and loss of several outcomes of interest such as local or distant recurrence, we did not feel those were disqualifying for our purposes because most of the variables that may affect the prognosis of patients, such as year of diagnosis, age, tumor location, size, grade and stage, and the outcome of interest (survival status and cause of death) in this study were all included.

### Covariates

Information of demographic variables including age, sex, race, and year of diagnosis, tumor characteristics including tumor location, size (maximal dimension), histological grade, and stage, treatment record including cancer-directed surgery or radiotherapy were all extracted, patients who had only received biopsy were included in no surgery group. The SEER database also provides data on the length of follow-up, patient’s vital status at the time of last follow-up, and the specific cause of death, enabling us to calculate the overall and disease-specific survival rate. Because of reporting omissions, data on tumor grade and size were not available in 126 cases (22.7%) and 65 cases (11.7%), respectively. To avoid potential bias, cases with missing data were excluded from respective univariate and multivariate analysis.

To investigate whether elderly and young patients have different survival risks, we converted the patient’s age to a categorical variable by 65 years old, which is often used as the threshold of elderly populations [21, 22]. Sex was divided into male and female. Race was divided into white, black, and American Indian/AK Native, Asian/Pacific Islander. Considering that the development of medical technology over time may have an impact on the prognosis, we divided the diagnosis year into two groups by 10 years. Tumor location was nonuniformly described in this database, and it is said tumors in non-extremity and deep location had better survival in patients with pleomorphic liposarcoma [7, 8]; thus categorical variables of soft tissue of extremity, soft tissue of axial, and internal tissue/organ were used based on available descriptors. Tumors in the arm or leg were classified as extremity, or as axial if they involved the trunk, pelvis, head, and neck. Internal tissue/organ referred to the mediastinum, peritoneum and retroperitoneum, viscera, bone, and spermatic cord. Tumor was categorized by 5 and 10 cm, which was commonly used to classify tumor size in soft tissue sarcomas [4, 23, 24]. Moreover, Cates also found that using 5 and 10 cm to classify tumor size was better than 5, 10, and 15 cm used in AJCC 8th staging system [25]. To avoid the potential bias from the multiple revisions in the TNM staging system, staging was determined by the SEER historic stage A system, which provides consistent definitions and continues to assign cases into local, regional, and distant disease over time.

### Statistical analyses

SEER*Stat version 8.3.6 (National Cancer Institute, Bethesda, MD, USA) was used to access the SEER database. The 5- and 10-year overall survival (OS) and disease-specific survival (DSS) were calculated with the Kaplan-Meier method. Log-rank test was applied to assess the effect of each demographic and clinicopathological variables on survival. Cox proportional hazards regression analysis was used to determine the hazard ratio (HR) of each covariate. Covariates with a *p* value < 0.1 on univariate analysis were entered into multivariate analysis. Specifically, age, site, tumor size, stage, surgery, and radiotherapy were examined as well as all two-way interactions. Schoenfeld residuals test [26] and Cox-Snell residuals [27] were applied to evaluate the proportional hazard assumption and the fitness of the Cox model, respectively. With the concern of competing risk of death in studies of elderly patients [28], we also extend the Cox regression to a Fine-Gray competing risk model by defining other causes of death as the competing event to reanalyze the prognostic significance of included variables [29]. All statistical analyses were performed using Stata/MP, version 14.0 (Stata Corporation, College Station, TX, USA), and *p* < 0.05 was considered statistically significant. All graphs were obtained using GraphPrism 7.0 software (GraphPad Software Inc., San Diego, CA, USA).

## Results

### Patient and tumor characteristics

Between 1996 and 2015, a total of 11410 cases of liposarcoma were diagnosed with histology confirmation, of which pleomorphic liposarcoma accounted for 7.8% (891/11410). Among them, 555 cases met our inclusion criteria, and all of these cases were pathologically diagnosed as pleomorphic liposarcoma (ICD-O-3-8854/3). Demographic and tumor characteristics of the entire study population were summarized in Table 1. The median age of the study cohort was 62 years (range 8 to 96 years). The sex distribution demonstrated a slight male predilection (*n*=331, 59.6%). Pleomorphic liposarcoma was most commonly happened in the soft tissue of extremity (*n*=317, 57.1%), followed by soft tissue of axial (*n*=156, 28.1%), and internal tissue/organ (*n*=82, 14.8%). The median tumor burden was 9.1 cm (range, 0.7–60 cm). Most patients had tumors in localized stage when firstly diagnosed (*n*=346, 62.3%). Almost all patients (*n*=501, 90.3%) received surgery specifically for this tumor, and radiation treatment was also received by a high proportion of patients (*n*=343, 61.8%).

**Table 1:** Demographic and clinical population characteristics of the study

Category	No. of patients	Category	No. of patients
Age (years)	^†^62 (8 to 96)	Size	^†^9.1 (0.7 to 60 )
< 65	300 (54.1%)	≤ 5 cm	115 (20.7%)
≥ 65	255 (45.9%)	5–10 cm	164 (29.6%)
Sex		> 10 cm	211(38.0%)
Male	331 (59.6%)	Unknown	65 (11.7%)
Female	224 (40.4%)	Stage	
Race		Localized	346 (62.3%)
White	443 (79.8%)	Regional	127 (22.9%)
Black	63 (11.4%)	Distant	57 (10.3%)
Other^‡^	46 (8.3%)	Unknown	25 (4.5%)
Unknown	3 (0.5%)	Cancer-directed Surgery	
Year of diagnosis		Performed	501 (90.3%)
1996 to 2005	221 (39.8%)	Not performed	51 (9.2%)
2006 to 2015	334 (60.2%)	Unknown	3 (0.5%)
Site		Radiotherapy	
Soft tissue of extremity	317 (57.1%)	Yes	343 (61.8%)
Soft tissue of axial	156 (28.1%)	No	177 (31.9%)
Internal tissue/organ	82 (14.8%)	Unknown	35 (6.3%)
Grade			
High (III/IV)	429(77.3%)		
Unknown	126(22.7%)		

^†^Median value with range in parentheses; Other^‡^, American Indian/AK Native, Asian/Pacific Islander

### Survivorship of patients with pleomorphic liposarcoma

**Fig. 2:**
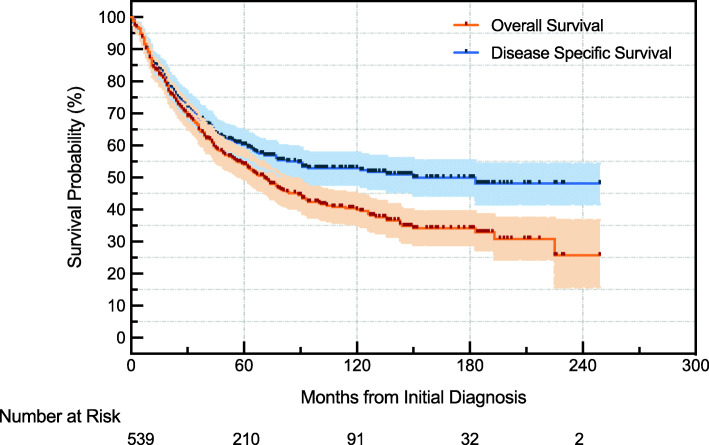
Kaplan-Meier plot of overall survival and disease specific survival for all patients

Survival across all individuals ranged from 0 to 249 months. Median follow-up was 100 months. OS for the entire series was 54% (95% confidence interval [CI], 49–58%) and 40% (95% CI, 35–45%) at 5 and 10 years, respectively (Fig. 2, Table 2). Disease-specific survival (DSS) for the entire series was 60% (95% CI, 56–65%) and 53% (95% CI, 48–58%) at 5 and 10 years, respectively (Fig. 2, Table 2). The DSS rate stabilized at about 10 years after the diagnosis and remained at about 50% at 20 years, while the OS rate continued to decline after 10 years and reached 26% at 20 years (Fig. 2). This finding suggested patients who survived the first 10 years after diagnosis were more likely to die of other causes rather than the pleomorphic liposarcoma itself.

**Table 2:** Survival stratified by demographic and clinical characteristics

Covariates	Overall Survival (95% CI)		Disease-specific survival (95% CI)	
	5-year	10-year	5-year	10-year
Entire series	54% (49–58%)	40% (35–45%)	60% (56–65%)	53% (48–58%)
Age (years)				
< 65	60% (54–66%)	53% (46–59%)	63% (56–68%)	55% (48–61%)
≥ 65	47% (40–53%)	26% (19–33%)	57% (50–64%)	51% (43–58%)
Site				
Soft tissue of extremity	57% (51–63%)	44% (38–50%)	64% (58–70%)	57% (50–63%)
Soft tissue of axial	53% (44–61%)	39% (29–48%)	58% (49–66%)	53% (43–62%)
Internal tissue/organ	43% (32–54%)	27% (16–38%)	49% (37–61%)	36% (24–49%)

*p values represent Kaplan-Meier log-rank tests for differences in survival by age and site; *CI* confidence interval

### Factors associated with survivorship

When examining DSS as a function of univariate analysis, age, sex, race, and year of diagnosis demonstrated no significant impact on DSS (Supplementary Table 1). Considering age is a common prognostic factor in various type of disease, we therefore also included it in the multivariate analysis. Interaction analysis found tumor size had significant interaction with tumor stage and radiotherapy (data not shown); thus, the interaction term size*stage and size*radiotherapy were both included in the final model. Therefore, the final Cox regression model included covariates of age, tumor site, size, stage, surgery, radiotherapy, and two interaction terms (size*stage, size*radiotherapy). Table 3 and Table 4 present the results of multivariate analysis.

**Table 3:** Multivariate analysis of disease specific survival

Covariates	Hazard ratio	95% CI	*p* value
Age (years)			
< 65	Reference group		
≥ 65	1.2	0.8–1.6	0.327
Site			
Soft tissue of extremity	Reference group		
Soft tissue of axial	0.8	0.5 to 1.2	0.253
Internal tissue/organ	1.0	0.6 to 1.6	0.964
Cancer-directed surgery			
Performed	Reference group		
Not performed	7.6	3.5 to 16.7	< 0.001
Size	–	–	–
Stage	–	–	–
Radiotherapy	–	–	–
Size*stage	–	–	–
Size*radiotherapy	–	–	–

Appropriate hazard ratios for the size*stage and size*radiotherapy are specified in Table 4; main effects of variables included in the interaction are not interpretable and therefore not provided; *CI* confidence interval

**Table 4:** Hazard ratios for disease-specific survival are presented by tumor stage and radiotherapy in different tumor size

Size	Stage	Hazard Ratio	95% CI	p value
≤ 5 cm	Localized	Reference group		
	Regional	6.5	1.8 to 22.7	0.004
	Distant	26.8	5.9 to 121.7	< 0.001
	Radiotherapy			
	No	Reference group		
	Yes	0.78	0.29 to 2.1	0.613
5–10 cm	Stage			
	Localized	Reference group		
	Regional	1.1	0.5 to 2.2	0.846
	Distant	3.5	1.4 to 9.1	0.009
	Radiotherapy			
	No	Reference group		
	Yes	0.4	0.2 to 0.9	0.028
> 10 cm	Stage			
	Localized	Reference group		
	Regional	1.1	0.7 to 1.9	0.630
	Distant	4.7	2.4 to 9.1	< 0.001
	Radiotherapy			
	No	Reference group		
	Yes	0.3	0.2 to 0.5	< 0.001

**Fig. 3:**
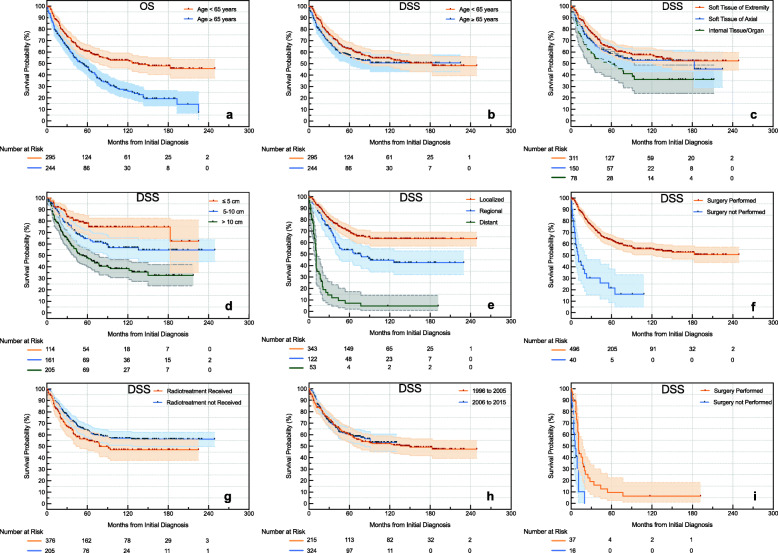
Kaplan-Meier plot stratified by clinical characteristics. **a** Overall survival (OS) stratified by age; disease-specific survival (DSS) stratified by **b** age, **c** tumor site, **d** tumor size, **e** stage, **f** surgery performed, **g** radiotherapy received, **h** year of diagnosis, and **i** DSS of patients in distant stage stratified by surgery performed

Figure 3a, b depicts the OS and DSS rate stratified by age at the time of diagnosis, respectively. Although the OS rate was significantly better in patients younger than 65 years old at 15 years follow-up (48% versus 20%, *p* < 0.001), and no statistically significant difference achieved in DSS rate between these two groups (51% versus 51%, *p* = 0.157). This result was also confirmed in multivariate analysis (Table 3).

The DSS rate, stratified according to anatomic location, is depicted in Fig. 3c. Patients with tumor in soft tissue of extremity had a significant better survival than in internal organs (52% vs 36%, *p* = 0.002) at 15 years follow-up. Patients with tumor in soft tissue of axial also had a DSS rate of 52% at 15 years follow-up, but there was no statistical difference in survival comparing to patients with tumor in internal organs (*p*= 0.076). In multivariate analysis, however, no survival difference was found among these three groups (Table 3).

The prognostic significance of tumor size can be interpreted from the Kaplan-Meier plot shown in Fig. 3d. Patients with tumor size at less than 5 cm, 5 to 10 cm, or larger than 10 cm had 15-year DSS rates of 75%, 55%, and 36%, respectively. All comparisons were statistically significant (*p* < 0.05).

Figure 3e depicts the DSS rate stratified by stage at the time of diagnosis. Patients with tumor in localized stage had the best survivorship compared to those in regional (63% versus 45%, *p* < 0.001) and distant stage (63% versus 5%, *p* < 0.001) at 10 years follow-up. When controlling for age, site, surgery, and radiotherapy, the association between DSS and tumor stage differed by tumor size (Table 4). Compared to patients in localized stage, regional stage predicted worse survival only when tumor size was less than 5 cm (HR = 6.5, 95%CI, 1.8 to 22.7, *p* = 0.004), while distant stage was a poor prognosis factor regardless of tumor size.

As to the effect of treatment paradigm, patients received surgery fared better 5-year DSS than patients without surgery treatment (63% versus 22%, HR = 7.6, 95%CI, 3.5–16.7, *p* < 0.001, Fig. 3f, Table 3). Almost all patients (97.4%) received beam radiation, and in univariate analysis, radiotherapy also provided survival benefits for patients with pleomorphic liposarcoma at 10 years follow-up (57% versus 47%, *p* < 0.05, Fig. 3g). However, after controlling for age, site, stage, and surgery, the relationship between DSS and radiotherapy depended on tumor size. Patients could only benefit from radiotherapy when the tumor was larger than 10 cm (HR = 0.3, 95%CI, 0.2 to 0.5, *p* < 0.001, Table 4, Table S5). Chemotherapy is divided into “yes” and “none/unknown” groups in SEER database, and the chemotherapy regimen is unknown; thus, it is impossible to compare the effects of chemotherapy on the prognosis of this tumor.

To evaluate the reason why internal tissue/organ lost effect on survival in the multivariate analysis. We compared the demographic and clinical characteristics stratified by tumor location (Supplementary Table 2). The analysis showed tumors happened in internal tissue/organ were more frequent with large tumor size (70.4% versus 36.6%), non-localized stage (66% versus 29%), and not received radiotherapy (59.1% versus 18.3%). All of these differences were significant (*p* < 0.05).

In addition, Schoenfeld residuals test results (Supplementary Table 3) supported that the Cox model meet the proportional hazard assumption, and Cox-Snell residuals plot (Supplementary Fig. 1) also demonstrated the cox model fit the data reasonably well. Finally, we regarded deaths not due to pleomorphic liposarcoma as competing events and extended the Cox regression model to evaluate competing risks of death (Supplementary Table 4, Supplementary Table 5). Except for the effect of radiotherapy on patients with tumors of 5–10 cm in size was lost, the *p* values of other parameters only got small changes.

## Discussion

Although general trends and characteristics of the clinical behavior of pleomorphic liposarcoma had been described in prior studies (Table 5), no large case series were available to verify these descriptions due to the exceeding rarity of this disease. To understand the outcomes of pleomorphic liposarcoma and potentially improve survival, a population-based registry with a long-term follow-up was used to identify prognostic factors significant in the survival of patients diagnosed with pleomorphic liposarcoma.

**Table 5:** Review of the literature: identified prognostic factors of pleomorphic liposarcoma

Author	Journal, year of publication^‡^	*N*^§^	Prognostic factor
Gebhard et al.^†^	AJSP, 2002	63	Age, truncal location, deep situation, size, vascular invasion, incomplete tumor excision; not grade and histology
Hornick et al.	AJSP, 2004	57	Age, central location, size, low mitotic rate, surgical margins and radiotherapy; not deep location and histology
Gardner et al.	AJSP, 2012	29	No statistical analysis
Oliveira et al.^†^	SDP, 2001	24	Upper extremities, size; not sex, age, histology, radiotherapy and chemotherapy
Downes et al.	MP, 2001	19	No statistical analysis

^†^Univariate analysis results; ^‡^*AJSP* The American Journal of Surgical Pathology, *MP* Modern Pathology, *SDP* Seminars in Diagnostic Pathology; ^*§*^*N* = number of patients

Our results regarding the 5-year OS rate substantiated the finding of prior studies, which was 54% in comparison to 57% and 63% in the two relatively large series [7, 8]. The 10-year survival rate was 40%, parallel to 39% reported by Zagars et al. [5]. We also found that the 5-year DSS rate was 60%, similar to 59% reported by Kimberly et al. [4]. The 10-year DSS rate was 53%, which was not reported previously. An interesting finding was the DSS rate started to plateau at about 10 years after initial diagnosis and remained at about 50% after 20 years (Fig. 2), indicating that patients survived the first 10 years after diagnosis were more likely to die of other causes that unrelated to pleomorphic liposarcoma, suggesting the regular follow-up time for this disease should at least be 10 years.

Some authors suggested most patients with pleomorphic liposarcoma were older adults (median age range 54–70 years), and it was extremely rare in patients under 22 years [6–9, 30]. This is consistent with our results that most of cases (*n* = 545, 98%) are above 22 years old, and the median age is 62 years. Young age (< 60 years) was recognized as a predictor of favorable outcome by Gebhard et al. [7] and Hornick et al. [8], but failed in an analysis reported by Oliveira et al. [9]. Our results indicate older age did have an adverse impact on OS, however, not in the disease-specific survival. This might be due to that elderly patients were more likely to have less chance to receive radical surgery or adjuvant treatments because of additional debilitating diseases, some of them might even die directly from those diseases so that it was impossible to observe the disease-specific death.

Current investigation indicates that sex and race have no impact on the survival of pleomorphic liposarcoma, which is consistent with prior findings [5, 9]. The presumption that the DSS for pleomorphic liposarcoma did not improve over the last 20 years is also reflected in Fig. 3h. This is logical because the treatment paradigm for pleomorphic liposarcoma did not change over the study period.

Site-related difference in the survival of pleomorphic liposarcoma had been previously described. Trunk tumors or those seated in deep (subfascial) location resulted in worse survival than those in extremity or superficial location. However, we cannot get a comparable analysis regarding the tumor depth, namely superficial, subfascial, intramuscular, or extra-compartmental, due to information about the specific location of the soft tissue is not available in the SEER database. Therefore, we classified the tumor site into soft tissue of extremity, axial, and internal tissues/organs based on available information. We found pleomorphic liposarcoma in internal tissues/organs presented worse outcome in univariate analysis but failed in multivariate analysis, which was similar to prior report, in which Hornick et al. [8] found deep location was also associated with decreased DSS but not in multivariate analysis. To investigate the potential causes of this finding, the demographics and tumor characteristic distribution stratified by tumor location was further studied. We found uneven distribution in tumor size, stage, and radiotherapy treatment. Comparing to tumor in internal tissue/organs, the significant higher portion of small size tumor in the soft tissue of extremity (28.1% versus 9.1%), more patients received radiotherapy (81.7% versus 40.9), and lower portion of advanced-stage tumor (29% versus 66%) might account for the apparently better survival in soft tissue pleomorphic liposarcoma in univariate analysis.

Large tumor size had been widely accepted as a predictor of poor prognosis in prior studies [8, 9, 12]. It was not surprising that our results also provided strong support to this finding. The correlation between large tumor size and worse survival might owing to that increased tumor size is likely a sign of more biologically active neoplasm and make it more difficult for surgeons to achieve safe surgical margins. Our results also supported the commonly held belief of that patients with distant stage disease survived much shorter than patients with disease in regional or localized stage. However, compared to patients in localized stage, regional stage predicted worse survival only when tumor size was less than 5 cm. This was rational because patients with larger tumor and in the regional stage were tended to receive radiotherapy and surgery (Supplementary Table 6). Thus, after controlling for these factors, the regional stage was not an independent prognostic factor when tumor size was greater than 5 cm.

As for the effect of treatment paradigm, surgery provided benefits to the survival of patients diagnosed with pleomorphic liposarcoma. We also found that 38 out 57 (67%) distant stage patients received surgery, but the reason why these patients received surgery were unknown due to the limited information SEER provided. With the concern that surgery might lose impact on survival in this group, we also did a subgroup analysis and found that surgery could still provide survival benefits to patients in distant stage in univariate analysis (Fig. 3i and Supplementary Table 7). The result was not surprising because several other studies also reported that surgery was associated with increased survival in patients presented with metastasis at diagnosis in soft tissue sarcoma [31, 32], but we had to mention that this result was not confirmed in multivariate analysis at the present study because only two distant stage patients receiving surgery had clear information on tumor size and radiotherapy record. Although the status of surgical margin is not accessible from the SEER database thus preventing us to get a further investigation about whether the clear surgical margin correlates with better survival, our data still supported that surgical resection was essential for pleomorphic liposarcoma. Multivariate analysis demonstrated radiotherapy was effective for patients with tumors larger than 5 cm (Table 4); however, only patients with tumor larger than 10 cm could benefit from radiotherapy at the competing risk model, which might own to a relatively large portion (30%) of patients in the 5–10 cm group died of events unrelated to pleomorphic liposarcoma. As most of the cases included in this study received post-surgery radiotherapy (data not shown), it is well known that large tumors always herald greater surgical challenges, which may lead to more positive surgical margins than small tumors. Therefore, using postoperative radiotherapy to kill residual tumor cells around the surgical margins may be more helpful for patients with large tumors. Previously, several studies reported that radiation treatment could improve survival of patients with myxoid liposarcoma and contribute to the local control of well-differentiated liposarcoma [33–36]. More recently, a randomized trial results also suggested that pre-operative radiotherapy might benefit patients with retroperitoneum liposarcoma [37]. Despite scarce evidence demonstrated the survival benefits of radiation treatment for pleomorphic liposarcoma, our results supported radiation should be an adjuvant therapy if feasible in patients with pleomorphic liposarcoma, especially in patients with large tumor size.

Limitations of this study are largely attribute to constraints inherent to the SEER database. First, pleomorphic liposarcoma is extremely rare and difficult to diagnosis because it lacks of immunohistochemical or molecular genetic features [11], while an inherent limitation of all SEER-based studies is the lack of central pathology review and uniformity of laboratory techniques in each registries SEER included, which may cause potential discrepancy of the histologic diagnosis. But we have tried to diminish the potential inaccuracies by only including patients with histological confirmation and excluding low grade tumors, which violated the common belief that pleomorphic liposarcoma is of high grade. Another concern is the lack of data on the use of chemotherapy. Although this information would undoubtedly provide important data to analyze as part of this investigation, it should not be considered a glaring deficit, as the use of chemotherapy for pleomorphic sarcoma is still undefined with current evidence [2, 7]. The incomplete data on two important clinical variables in this study might also raise some bias. The anatomic location and grade of disease were not reported in 11.7% and 22.7% of the study cohorts. To minimize the potential effect of these incomplete data, we have excluded cases with missing information in the current investigation.

Despite the aforementioned limitations, to our knowledge, this is the first time of using SEER data, which included a large number of patients and long-term follow-up, to analyze pleomorphic liposarcoma specifically. We believe our study constitutes a substantial step toward identifying the survival outcome and corresponding prognostic factors of patients diagnosed with pleomorphic liposarcoma. We found older age was associated with worse OS but not DSS, indicating age was not an independent factor for the death of pleomorphic liposarcoma. Our results are also helpful for both patients and their treating specialists to know that a 10-year follow-up is needed because pleomorphic liposarcoma is unlikely to be the ultimate cause of death after surviving more than 10 years. Radiotherapy is recommended if patients have a pleomorphic liposarcoma larger than 10 cm in size. New therapeutic options despite surgery and radiation still need to improve the benefit of current treatment paradigm.

## Supplementary Information

**Additional file 1 Table S1.** Univariate Analysis of Disease Specific Survival**. Table S2.** Clinical characteristics of patients with pleomorphic liposarcoma stratified by tumor location**. Table S3.** Proportional hazard assumption test by Schoenfeld residuals**. Table S4.** Competing Risk Regression**. Table S5.** Hazard ratios for Disease Specific Survival are Presented by Tumor Stage and Radiotherapy in Different Tumor Size**. Table S6.** Clinical characteristics of patients with pleomorphic liposarcoma stratified by tumor size and stage**. Table S7.** Clinical characteristics of patients with pleomorphic liposarcoma in distant stage stratified by surgery**. Fig. S1** Cox-Snell residual plot. The Cox-Snell residual plot suggests the proportional hazards assumption holds, and the multivariate Cox proportional hazard model fit the data reasonably well.

## Data Availability

The raw data of the case cohort was uploaded to the *Journal of Orthopaedic Surgery and Research.*

## Abbreviations

ICD-O-3: International Classification of Diseases for Oncology, Third Edition
CI: Confidence interval
SEER: Surveillance Epidemiology and End Results database
OS: Overall survival
DSS: Disease-specific survival (DSS)
HR: Hazard ratio
