# Correction to: Survivorship and prognostic factors for pleomorphic liposarcoma: a population-based study

**DOI:** 10.1186/s13018-021-02369-7

**Published:** 2021-03-29

**Authors:** Lu Wan, Chao Tu, Lin Qi, Zhihong Li

**Affiliations:** 1 Department of Orthopedics, The Second Xiangya Hospital, Central South University, 139 Renming Road, Changsha, 410011 Hunan People’s Republic of China; 2 Vaccine and Immunotherapy Center, Infectious Diseases Division, Department of Medicine, Massachusetts General Hospital and Harvard Medical School, 149 13th Street, Charlestown, MA 02129 USA; 3 Key Laboratory of Tumor Models and Individualized Medicine, The Second Xiangya Hospital, Central South University, 139 Renming Road, Changsha, 410011 Hunan People’s Republic of China

**Correction to: J Orthop Surg Res 16, 175 (2021)**

**https://doi.org/10.1186/s13018-021-02327-3**

Following the publication of the original article [1], author noticed that the email addresses of 2 authors are captured in the correspondence field. Lu Wan and Chao Tu are not co-corresponding authors for this paper. Hence, correspondence symbol has been deleted from the author group as well (as shown above).

The original article has been corrected.
