# Supplementary material for: Survivorship and prognostic factors for pleomorphic liposarcoma: a population-based study
Source: J Orthop Surg Res. 2021 Mar 4;16:175. doi: 10.1186/s13018-021-02327-3 (PMC7931523; doi:10.1186/s13018-021-02327-3)
Supplement: Supplementary file 1 — Additional file 1 Table S1. Univariate Analysis of Disease Specific Survival. Table S2. Clinical characteristics of patients with pleomorphic liposarcoma stratified by tumor location. Table S3. Proportional hazard assumption test by Schoenfeld residuals. Table S4. Competing Risk Regression. Table S5. Hazard ratios for Disease Specific Survival are Presented by Tumor Stage and Radiotherapy in Different Tumor Size. Table S6. Clinical characteristics of patients with pleomorphic liposarcoma stratified by tumor size and stage. Table S7. Clinical characteristics of patients with pleomorphic liposarcoma in distant stage stratified by surgery. Fig. S1 Cox-Snell residual plot. The Cox-Snell residual plot suggests the proportional hazards assumption holds, and the multivariate Cox proportional hazard model fit the data reasonably well. [file 13018_2021_2327_MOESM1_ESM.docx]

| **Table S1. Univariate Analysis of Disease Specific Survival** | | | |
| --- | --- | --- | --- |
| Covariates | Hazard Ratio | 95% CI | p value |
| Age *(versus < 65 years*) |  |  |  |
| ≥ 65 | 1.2 | 0.9 to 1.6 | 0.160 |
| Sex (*versus Female*) |  |  |  |
| Male | 0.93 | 0.7 to 1.2 | 0.584 |
| Race (*versus* *White*) |  |  | 0.463 |
| Black | 1.2 | 0.8 to 1.9 | 0.387 |
| Other* | 0.8 | 0.5 to 1.4 | 0.512 |
| Year of Diagnosis (*versus* *1996-2005*) |  |  |  |
| 2006-2015 | 0.9 | 0.7 to 1.1 | 0.320 |
| Site (*versus* *Soft tissue of extremity*) |  |  | < 0.001 |
| Soft tissue of axial | 1.2 | 0.9 to 1.7 | 0.213 |
| Internal tissue/organ | 1.7 | 1.2 to 2.5 | 0.002 |
| Size (*versus* ≤ *5cm*) |  |  |  |
| 5-10 cm | 1.7 | 1.1 to 2.7 | < 0.025 |
| > 10 cm | 3.0 | 2.0 to 4.6 | < 0.001 |
| Stage (*versus* *Localized*) |  |  | < 0.001 |
| Regional | 1.7 | 1.3 to 2.4 | 0.001 |
| Distant | 8.7 | 6.1 to 12.5 | < 0.001 |
| Surgery (*versus* *Performed*) |  |  |  |
| No | 4.8 | 3.2 to 7.2 | < 0.001 |
| Radiotherapy (*versus Performed*) |  |  |  |
| No | 1.3 | 1.0 to 1.8 | 0.05 |
| *American Indian/AK Native, Asian/Pacific Islander; CI = confidence interval | | | |

| **Table S2. Clinical characteristics of patients with pleomorphic liposarcoma stratified by tumor location** | | | |
| --- | --- | --- | --- |
| Category | Soft tissue of extremity | Internal tissue/organ | *p |
| Age (years) |  |  | 0.490 |
| < 65 | 130 (58%) | 28 (64%) |  |
| ≥ 65 | 94 (42%) | 16 (36%) |  |
| Size |  |  | < 0.001 |
| ≤ 5 cm | 63 (28%) | 4 (9%) |  |
| 5-10 cm | 79 (35%) | 9 (21%) |  |
| > 10 cm | 82 (37%) | 31(70%) |  |
| Stage |  |  | < 0.001 |
| Localized | 159 (71%) | 15 (34%) |  |
| Regional | 52 (23%) | 20 (46%) |  |
| Distant | 13 (6%) | 9 (20%) |  |
| Cancer-directed Surgery |  |  | 1.0 |
| Performed | 218 (97%) | 43 (98%) |  |
| Not performed | 6 (3%) | 1 (2%) |  |
| Radiotherapy |  |  | < 0.001 |
| Yes | 183 (82%) | 18 (41%) |  |
| No | 41 (18%) | 26 (59%) |  |
| * p values represent chi-squared test or Fisher-exact test for differences in clinical characteristics by site | | | |

| **Table S3. Proportional hazard assumption test by Schoenfeld residuals** | | | | |
| --- | --- | --- | --- | --- |
| Category | Rho | Chi2 | df | p value |
| Age | -0.124 | 2.33 | 1 | 0.127 |
| Site |  |  |  |  |
| Soft tissue of extremity | - | - | 1 | - |
| Soft tissue of axial | -0.126 | 2.58 | 1 | 0.108 |
| Internal tissue/organ | 0.004 | 0.00 | 1 | 0.953 |
| Size | -0.004 | 0.01 | 1 | 0.923 |
| ≤ 5 cm | - | - | 1 | - |
| 5-10 cm | 0.016 | 0.04 | 1 | 0.838 |
| > 10 cm | 0.010 | 0.02 | 1 | 0.900 |
| Stage |  |  |  |  |
| Localized | - | - | 1 | - |
| Regional | -0.137 | 2.67 | 1 | 0.102 |
| Distant | -0.007 | 0.01 | 1 | 0.920 |
| Cancer-directed Surgery | -0.003 | 0.00 | 1 | 0.962 |
| Radiotherapy | 0.085 | 1.07 | 1 | 0.301 |
| Size (≤ 5 cm) * Stage (Localized) | - | - | 1 | - |
| Size (≤ 5 cm) * Stage (Regional) | - | - | 1 | - |
| Size (≤ 5 cm) * Stage (Distant) | - | - | 1 | - |
| Size (5-10 cm ) * Stage (Localized) | - | - | 1 | - |
| Size (5-10 cm) * Stage (Regional) | 0.083 | 1.01 | 1 | 0.316 |
| Size (5-10 cm) * Stage (Distant) | -0.061 | 0.60 | 1 | 0.439 |
| Size (> 10 cm) * Stage (Localized) | - | - | 1 | - |
| Size (> 10 cm) * Stage (Regional) | 0.101 | 1.46 | 1 | 0.227 |
| Size (> 10 cm) * Stage (Distant) | 0.007 | 0.01 | 1 | 0.921 |
| Size (≤ 5 cm ) * Radiotherapy (Yes) | - | - | 1 | - |
| Size (≤ 5 cm ) * Radiotherapy (No) | - | - | 1 | - |
| Size (5-10 cm ) * Radiotherapy (Yes) | - | - | 1 | - |
| Size (5-10 cm ) * Radiotherapy (No) | -0.062 | 0.58 | 1 | 0.445 |
| Size (> 10 cm ) * Radiotherapy (Yes) | - | - | - | - |
| Size (> 10 cm ) * Radiotherapy (No) | -0.094 | 1.32 | 1 | 0.251 |
| Global test |  | 12.52 | 15 | 0.639 |

| **Table S4. Competing Risk Regression** | | | |
| --- | --- | --- | --- |
| Covariates | Hazard Ratio | 95% CI | p value |
| Age (*versus < 65 years*) |  |  |  |
| ≥ 65 | 1.1 | 0.8 to 1.5 | 0.697 |
| Site (*versus* *Soft tissue of extremity*) |  |  |  |
| Soft tissue of axial | 0.8 | 0.5 to 1.3 | 0.375 |
| Internal tissue/organ | 0.9 | 0.6 to 1.5 | 0.791 |
| Surgery (*versus Performed*) |  |  |  |
| No | 7.4 | 3.5 to 15.3 | < 0.001 |
| Size | - | - | - |
| Stage | - | - | - |
| Radiotherapy | - | - | - |
| Size*Stage | - | - | - |
| Size*Radiotherapy | - | - | - |
| Appropriate hazard ratios for the size*stage and size*radiotherapy are specified in Table S5; main effects of variables included in the interaction are not interpretable and therefore not provided; CI = confidence interval | | | |

| **Table S5. Hazard ratios for Disease Specific Survival are Presented by Tumor Stage and Radiotherapy in Different Tumor Size** | | | | |
| --- | --- | --- | --- | --- |
| Size | Stage | Hazard Ratio | 95% CI | p value |
| ≤ 5 cm | Localized | Reference group |  |  |
|  | Regional | 6.6 | 1.7 to 25.2 | 0.006 |
|  | Distant | 27.3 | 9.6 to 77.7 | < 0.001 |
|  | Radiotherapy |  |  |  |
|  | No | Reference group |  |  |
|  | Yes | 0.77 | 0.3 to 1.9 | 0.580 |
| 5-10 cm | Stage |  |  |  |
|  | Localized | Reference group |  |  |
|  | Regional | 1.1 | 0.6 to 2.2 | 0.742 |
|  | Distant | 3.2 | 1.1 to 9.2 | 0.028 |
|  | Radiotherapy |  |  |  |
|  | No | Reference group |  |  |
|  | Yes | 0.6 | 0.3 to 1.3 | 0.196 |
| > 10 cm | Stage |  |  |  |
|  | Localized | Reference group |  |  |
|  | Regional | 1.1 | 0.7 to 1.7 | 0.772 |
|  | Distant | 4.8 | 2.8 to 8.1 | < 0.001 |
|  | Radiotherapy |  |  |  |
|  | No | Reference group |  |  |
|  | Yes | 0.4 | 0.2 to 0.6 | < 0.001 |

| **Table S6. Clinical characteristics of patients with pleomorphic liposarcoma stratified by tumor size and stage** | | | | | | | |
| --- | --- | --- | --- | --- | --- | --- | --- |
| Category | Size > 5cm | | |  | Size ≤ 5cm | | |
|  | Localized | Regional | *p |  | Localized | Regional | *p |
| Age (years) |  |  | 0.777 |  |  |  | 1.000 |
| < 65 | 98 (60%) | 56 (58%) |  |  | 38 (48%) | 3 (43%) |  |
| ≥ 65 | 65 (40%) | 40 (42%) |  |  | 42 (52%) | 4 (57%) |  |
| Site |  |  | < 0.001 |  |  |  | 0.742 |
| Soft tissue of extremity | 104 (64%) | 46 (48%) |  |  | 55 (69%) | 6 (86%) |  |
| Soft tissue of axial | 47 (29%) | 30 (31%) |  |  | 22 (27%) | 1 (14%) |  |
| Internal tissue/organ | 12 (7%) | 20 (21%) |  |  | 3 (4%) | 0 (0%) |  |
| Surgery |  |  | 0.049 |  |  |  | NA |
| Yes | 156 (96%) | 96 (100%) |  |  | 80 (100%) | 7 (100%) |  |
| No | 7 (4%) | 0 (0%) |  |  | 0 (0%) | 0 (0%) |  |
| Radiotherapy |  |  | < 0.001 |  |  |  | 0.215 |
| Yes | 134 (82%) | 60 (63%) |  |  | 55 (69%) | 3 (43%) |  |
| No | 29 (18%) | 36 (37%) |  |  | 25 (31%) | 4 (57%) |  |
| * p values represent chi-squared test or Fisher-exact test for differences in clinical characteristics by size and stage | | | | | | | |

| **Table S7. Clinical characteristics of patients with pleomorphic liposarcoma in distant stage stratified by surgery** | | | |
| --- | --- | --- | --- |
| Category | Surgery received | Surgery not received | p |
| Age (years) |  |  | *0.260 |
| < 65 | 20 (53%) | 7 (37%) |  |
| ≥ 65 | 18 (47%) | 12 (63%) |  |
| Size |  |  | *0.037 |
| ≤ 5 cm | 4 (11%) | 0 |  |
| 5-10 cm | 10 (26%) | 4 (21%) |  |
| > 10 cm | 19 (50%) | 6(32%) |  |
| Unknown | 5 (13%) | 9 (47%) |  |
| Site |  |  | *0.443 |
| Soft tissue of extremity | 15 (40%) | 6 (32%) |  |
| Soft tissue of axial | 8 (21%) | 7 (36%) |  |
| Internal tissue/organ | 15 (39%) | 6 (32%) |  |
| Radiotherapy |  |  | *< 0.001 |
| Yes | 19 (50%) | 4 (21%) |  |
| No | 19 (50%) | 0 |  |
| Unknown | 0 | 15 (79%) |  |
| 5-year DSS | ^†^10% (2%-22%) | 0 | ^‡^0.002 |
| * p values represent chi-squared test or Fisher-exact test for differences in clinical characteristics stratified by surgery; ^†^ DSS rate with 95% CI in parentheses; ^‡^p value of log-rank test for survival difference. | | | |


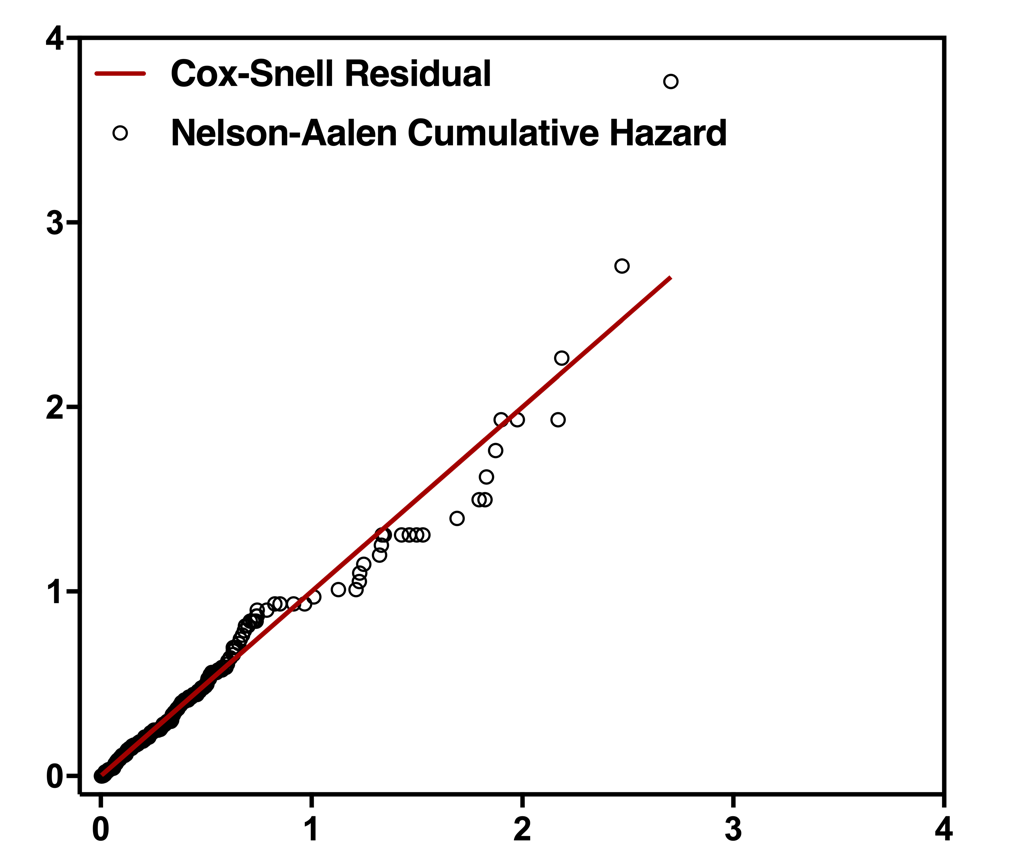
**Fig. S1** ﻿Cox-Snell residual plot. The Cox-Snell residual plot suggests the proportional hazards assumption holds, and the multivariate Cox proportional hazard model fit the data reasonably well.
